# Carbohydrate binding module-fused antibodies improve the performance of cellulose-based lateral flow immunoassays

**DOI:** 10.1038/s41598-021-87072-7

**Published:** 2021-04-12

**Authors:** Adrian Elter, Tina Bock, Dieter Spiehl, Giulio Russo, Steffen C. Hinz, Sebastian Bitsch, Eva Baum, Markus Langhans, Tobias Meckel, Edgar Dörsam, Harald Kolmar, Gerhard Schwall

**Affiliations:** 1 Institute for Organic Chemistry and Biochemistry, Technical University of Darmstadt, Alarich-Weiss-Strasse 4, 64287 Darmstadt, Germany; 2 Merck Lab, Technical University of Darmstadt, Alarich-Weiss-Strasse 8, 64287 Darmstadt, Germany; 3 Sustainability, Science and Technology Relations, Merck KGaA, Frankfurter Strasse 250, 64293 Darmstadt, Germany; 4 Institue of Printing Science and Technology, Technical University of Darmstadt, Magdalenenstrasse 2, 64289 Darmstadt, Germany; 5 Department of Biotechnology, Technical University of Braunschweig, Spielmannstrasse 7, 38124 Braunschweig, Germany; 6 Abcalis GmbH, Inhoffenstrasse 7, 38124 Braunschweig, Germany; 7 Macromolecular Chemistry and Paper Chemistry, Technical University of Darmstadt, Alarich-Weiss-Strasse 8, 64287 Darmstadt, Germany

**Keywords:** Biochemistry, Biotechnology, Molecular biology

## Abstract

Since the pandemic outbreak of Covid-19 in December 2019, several lateral flow assay (LFA) devices were developed to enable the constant monitoring of regional and global infection processes. Additionally, innumerable lateral flow test devices are frequently used for determination of different clinical parameters, food safety, and environmental factors. Since common LFAs rely on non-biodegradable nitrocellulose membranes, we focused on their replacement by cellulose-composed, biodegradable papers. We report the development of cellulose paper-based lateral flow immunoassays using a carbohydrate-binding module-fused to detection antibodies. Studies regarding the protein binding capacity and potential protein wash-off effects on cellulose paper demonstrated a 2.7-fold protein binding capacity of CBM-fused antibody fragments compared to the sole antibody fragment. Furthermore, this strategy improved the spatial retention of CBM-fused detection antibodies to the test area, which resulted in an enhanced sensitivity and improved overall LFA-performance compared to the naked detection antibody. CBM-assisted antibodies were validated by implementation into two model lateral flow test devices (pregnancy detection and the detection of SARS-CoV-2 specific antibodies). The CBM-assisted pregnancy LFA demonstrated sensitive detection of human gonadotropin (hCG) in synthetic urine and the CBM-assisted Covid-19 antibody LFA was able to detect SARS-CoV-2 specific antibodies present in serum. Our findings pave the way to the more frequent use of cellulose-based papers instead of nitrocellulose in LFA devices and thus potentially improve the sustainability in the field of POC diagnostics.

## Introduction

Low-instrumented point-of-care (POC) devices became a powerful tool for the rapid and inexpensive analysis of clinical parameters, as well as for the frequent monitoring of food safety and environmental conditions^1,2^. POC diagnostic devices often obviate the need of centralized laboratories and provide immediate qualitative and semi-quantitative results, independent of further technical equipment^3^. The increasing demand for inexpensive POC devices is mainly driven by the continuous growth of the world population linked to immense healthcare challenges including persistent and newly emerging pandemics like tuberculosis, HIV/AIDS and COVID-19. In September 2020, the U. S. Food and Drug Administration issued the emergency authorization for the first COVID-19 serological POC test, enabling the detection of antibodies originating from a prior infection with SARS-CoV-2 using fingertip blood samples^4^. In 2024 the global market of POC diagnostics is projected to reach more than 46 billion USD, while the segment of lateral flow assays (LFAs) accounts for the largest proportion^5,6^. Low production costs and the ease of applicability resulted in the development and implementation of LFAs in numerous fields of application. LFAs are continuously used in hospitals, medical practices and clinical laboratories, making it possible to detect defined antigens, including bacterial and viral-derived proteins, hormones, antibodies, DNA, RNA, and other substances obtained by various patient-derived samples like urine, serum, whole blood and others^7^. Common lateral flow assays are composed of a nitrocellulose membrane and conjugate- and adsorption-pads, typically reinforced by plastic backings and plastic housing^8^. Monoclonal antibodies, usually derived from rodents and produced in mammalian cell culture, enable the detection of analytes in an immunochromatographic manner, while the nitrocellulose membrane functions as the matrix on which the antibody-analyte complex forms and retention of these complexes takes place. In most cases, accumulation of colored antibody-nanoparticle conjugates bound by an analyte-antibody complex to the test-area contributes to the convenient visual readout by the user^8,9^.

The overall production cost of LFAs is dominated by the generation of detection antibodies, the plastic housing and the nitrocellulose membrane. Beside the housing, which does not necessarily contribute to the functional unit of an LFA, researchers and companies focused on the substitution of nitrocellulose (NC) membranes by inexpensive cellulose papers and the cost reduction of detection antibodies^10,11^. The absorbent- and sample-pad are typically made of cellulose paper and thus account only to one tenth of the cost in comparison to their nitrocellulose counterpart^12^. However, in some cases there is a need of sample-pads made of glass fibre or synthetic fibres enabling the separation of plasma from whole blood^13^. Regarding the need of monoclonal antibodies, switching the expression system from mammalian cell culture to bacterial or insect protein expression could accelerate the antibody production and further reduce the overall cost of an LFA^14,15^. In addition to the possibility of cost reduction, replacement of NC by cellulose-based materials results in a simplified LFA production (roll to roll manufacturing) and a favorable sustainability index, since cellulose-based materials display excellent biocompatibility and biodegradability^10,16,17^.

Beside other characteristics regarding the complexity, user considerations, sample preparation, maintenance, reproducibility and user-friendly result interpretation, the POC device must ensure the sensitive detection of specified parameters. Some of these criteria, including result interpretation and sensitivity are directly related to the immobilization and spatial retention of detection agents within the test aera^8^. The convenient non-covalent immobilization of antibodies and other proteins on NC membranes caused constant overlooking of the possibility of replacing NC by other cellulose-composed papers. Reported approaches concerning the covalent immobilization of compounds onto cellulose-fibers represent promising tools to improve paper-based analytic devices, however nitrocellulose-composed LFAs still dominate the market^18^. Interestingly, the already favorable binding capacity of proteins onto NC membranes can be further improved by fusion with a nitrocellulose-binding protein^19^. Inspired by these findings, we assumed that a similar approach could result in improved binding capacity of proteins onto cellulose-based paper.

Several fungi and bacteria are capable of degrading cellulose as a source of energy conversion using carbohydrate-active enzymes and enzyme complexes (cellulosomes). The carbohydrate-binding module (CBM) represents an important component of the enzyme apparatus. These multimeric protein structures often possess one or several CBM domains improving the biomass conversion by constant substrate supply^20,21^. A variety of CBMs are known, displaying different binding properties and substrate specificities^20,22^. Recently, a study addressing the applicability of a CBM-fused ZZ-domain for the colorimetric detection of DNA strands on cellulose particles was published by Rosa et al.^23^. Additionally, a recently published study addressed the applicability of protein A-derived domains fused to different CBM domains, resulting in improved binding onto cellulose-paper used for lateral flow immuno assays^24^. However, this newly developed platform displayed several limitations including unwanted cross-reactivity to (detection-) antibodies from different species. Moreover, the presence of antibodies in human serum excludes the application of Protein A-derived domains fused to CBM domains in a serological LFA setup.

**Figure 1:**
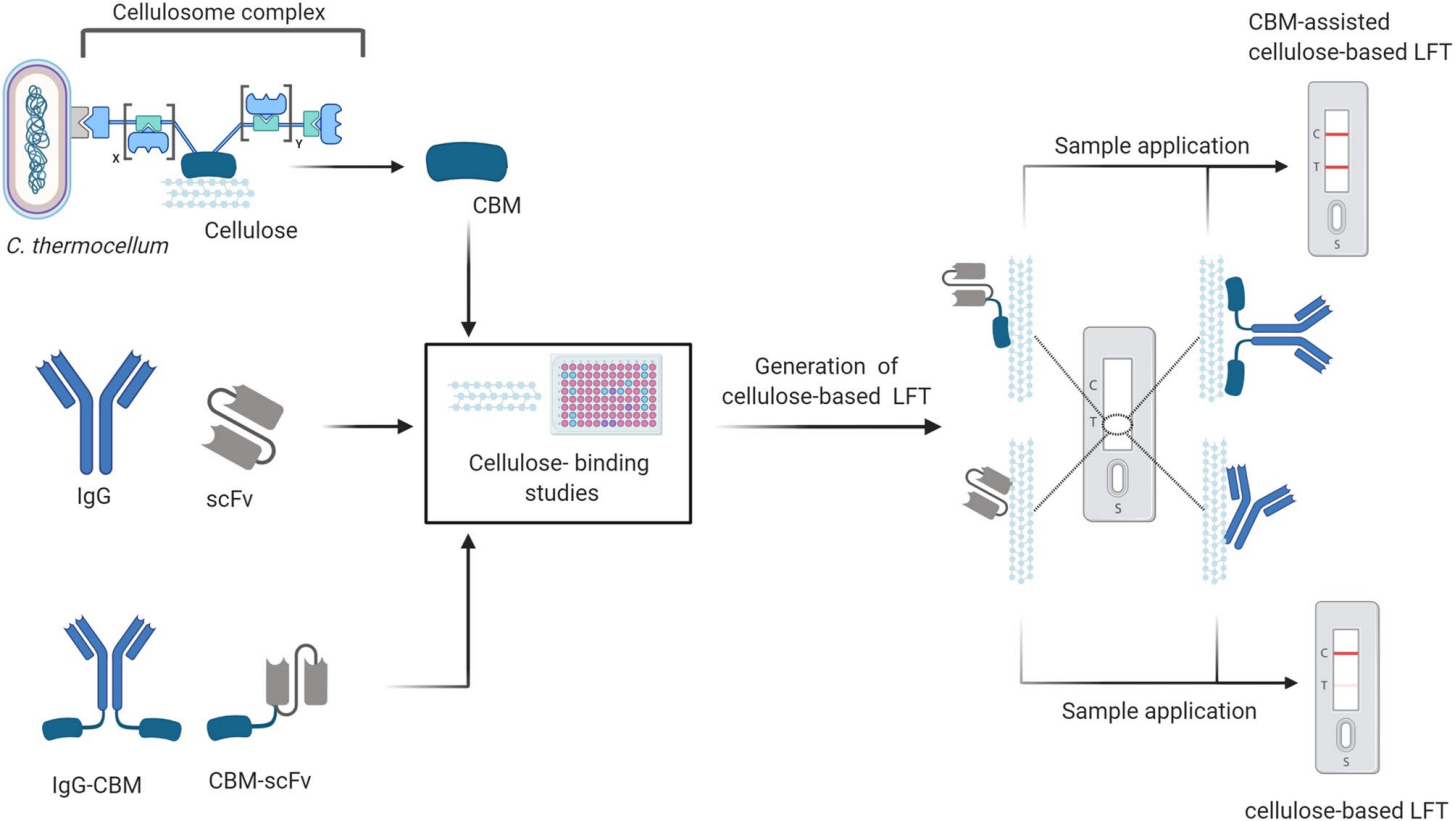
Workflow of the generation of carbohydrate-binding module-assisted lateral flow assays: The carbohydrate-binding module 3a of *Clostridium thermocellum* (CBM) was fused to different single chain variable Fragments (scFv) or to full-length antibodies (IgG). The resulting CBM-fusion proteins, as well as the non-fused CBM, scFv and full-length antibody were examined regarding their binding properties towards cellulose paper. Cellulose-based lateral flow assays were performed and compared using the CBM-fused antibodies as well as the solitary antibodies.

In this study, we focused on the carbohydrate-binding module 3a (CBM3a), contributing to the cellulosomal scaffold subunit of *Clostridium thermocellum*^25^. We assumed that the genetic fusion of single-chain variable fragments (scFv) or full-length antibodies to the CBM3a domain could result in an improved cellulose-binding capacity, and thus leading to an enhanced sensitivity and overall performance of cellulose-based lateral flow devices, compared to solitary scFvs or full-length antibodies. Cellulose and nitrocellulose binding studies disclosed important findings about the binding capacity of CBM and CBM-fused molecules. We examined the applicability in the context of cellulose-based LFAs using CBM-fused detection antibodies based on a hCG-specific scFv (CBM-anti-hCG scFv)^26^, a Fc-specific scFv (CBM-anti-Fc scFv)^27^, both produced in *E.coli* and on a full-length IgG CBM-fused anti-human-IgG multiclonal mixture (anti-hIgG-CBM), derived from an animal-free phage-display antibody discovery platform^28^ (Fig. 1).

Here we report the applicability of CBM-fused antibodies for the facile development of cellulose-based lateral flow assays for pregnancy testing. Encouraged by our findings, we applied our system for the detection of Sars-CoV-2-specific IgGs present in serum. Furthermore, our results demonstrate an improvement in sensitivity and a convenient result interpretation compared to a non-CBM-assisted cellulose-based LFA. Since we fused the detection antibodies directly to the CBM domain, our newly developed platform could enable the direct substitution of nitrocellulose membranes in established lateral flow test setups by cellulose-based papers, thereby also including serological LFAs. With respect to the worldwide increasing demand of POC diagnostics, our generic approach enables the replacement of nitrocellulose by biodegradable cellulose, which could result in an improved sustainability of innumerable lateral flow test devices as well as in a reduction in manufacturing costs.

## Results

### Generation of CBM-fused and solitary detection antibodies and antibody fragments

The DNA sequence coding for the carbohydrate-binding module 3a of *Clostridium thermocellum* (CBM) was used to generate fusion genes encoding CBM-anti-hCG scFv and CBM-anti-Fc scFv. Both scFvs were derived from chicken immunization and yeast display library screening and were described in previous studies^26,27^. Likewise, to serve as a control anti-hCG scFv and the solitary anti-Fc scFv were produced in *E. coli* via gene expression using the pET30 expression vector. All genetic constructs exhibit a C-terminal sequence coding for a StrepII-tag^29^. All proteins were successfully produced in *E. coli* T7 Shuffle Express and were subsequently purified using a Strep-Tactin superflow high capacity cartridge. SDS-PAGE analysis revealed sufficient purity (Figure S1). Furthermore, we generated a C-terminal CBM fusion to the mouse IgG2a heavy chains of different recombinant monoclonal antibodies anti-human IgG. The resulting mIgG2a-CBM multiclonal mixture (anti-hIgG, anti-hIgG-CBM) recognizes all subclasses of human Immunoglobulin G. Both, CBM-fused as well as the corresponding IgG antibodies only were successfully produced in Expi293F mammalian cells. After protein A purification, SDS-PAGE analysis confirmed sufficient protein purity (Figure S2).

### Cellulose and nitrocellulose binding studies

**Figure 2:**
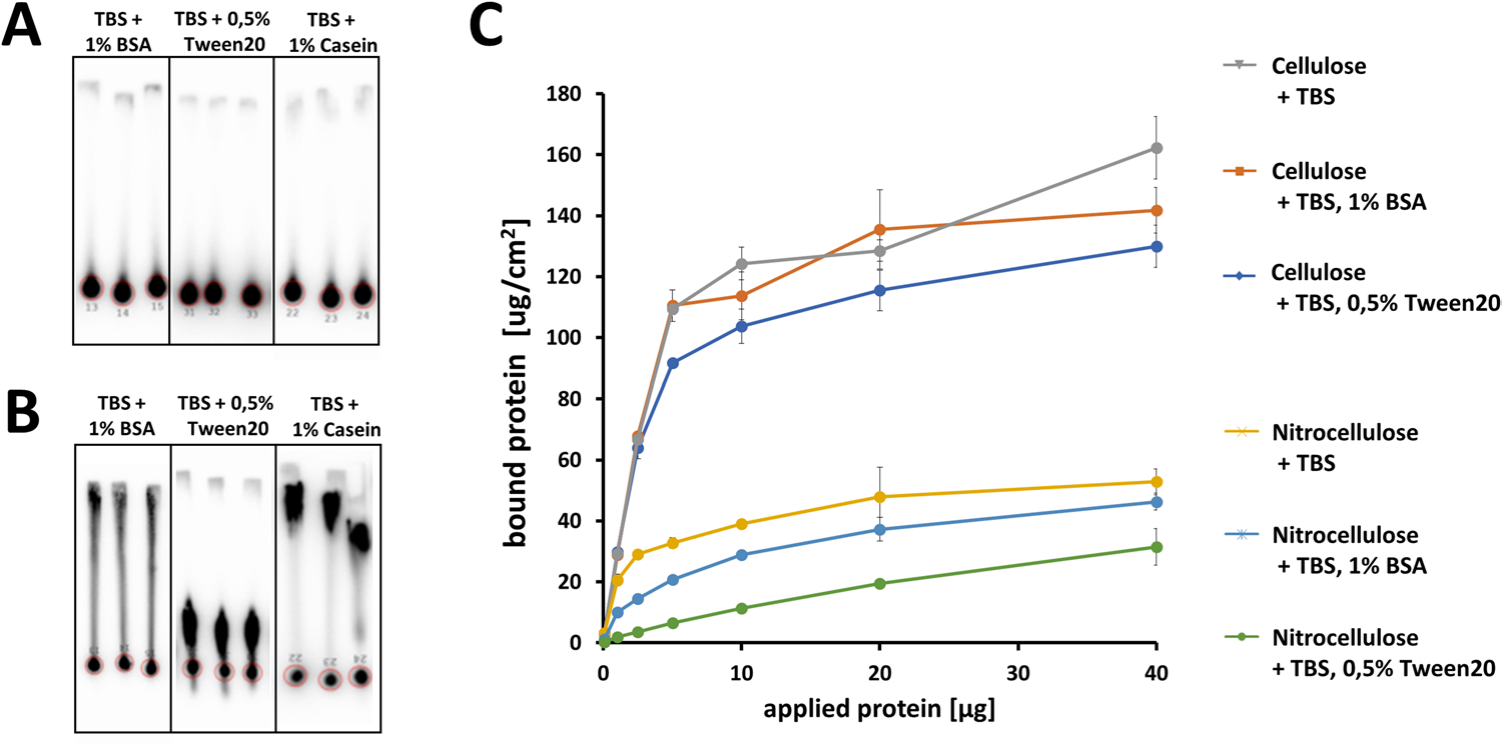
Cellulose and nitrocellulose binding studies using CBM protein, determining the influence of different buffer additives. ^125^I radiolabeled CBM protein was applied to (**A**) cellulose-paper stripes or (**B**) nitrocellulose membrane. After drying, application of buffer solution containing different additives, protein localization and quantitative analysis of bound and washed-off protein was analyzed using phosphor imaging (Figure S3). (**C**) Quantitative analysis of protein-binding towards cellulose and nitrocellulose was performed according to Figure S4. Standard derivation was calculated using data derived from experimental triplicates.

In order to examine the protein binding characteristics, varying amounts of isotope-labeled CBM protein were spotted onto nitrocellulose membranes or cellulose paper. After a wash-off procedure using different buffer solutions, a radiogram analysis revealed the spatial retention of CBM onto cellulose paper (Fig. 2A), while in case of nitrocellulose a significant proportion of CBM protein was washed away in the direction of the lateral flow, with only a minor fraction remaining in the area contributing to the test line of common lateral flow test devices (Fig. 2B). Since common lateral flow test devices contain different buffer additives, the influence on cellulose binding was investigated using wash buffer containing either 1% (w/v) BSA or 0.5% (w/v) Tween20. The quantitative analysis demonstrated an enhanced binding of CBM on cellulose paper compared to the NC membrane, independently of the wash buffer additives (Fig. 2C). When comparing the amounts of residual ^125^I radiolabeled CBM protein in the membrane spot where first applied before wash off, the CBM protein demonstrated superior binding to cellulose, resulting in an approximately threefold amount of protein, remaining on the initial application area. In case of nitrocellulose experiments, buffer additives, like 1% (w/v) BSA or 0.5% (w/v) Tween20 resulted in significant increase of the washed-off protein proportion (Fig. 2C).

**Figure 3:**
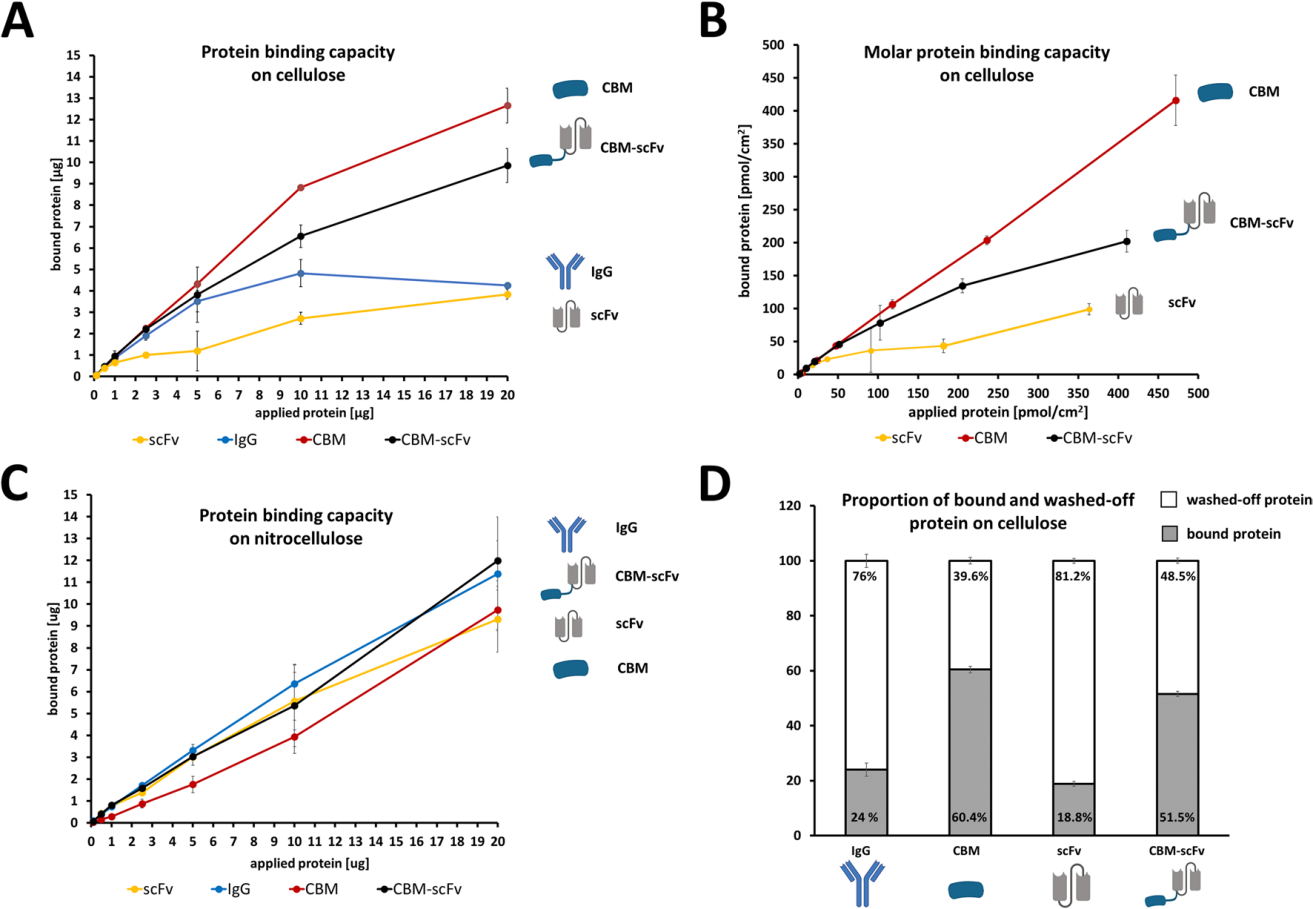
Cellulose and nitrocellulose protein-binding-studies using following proteins: CBM, scFv, CBM-scFv and IgG. (**A**) Protein binding capacity on cellulose paper. (**B**) Molar protein binding capacity on cellulose. (**C**) Protein binding capacity on nitrocellulose. (**D**) Proportions of bound and washed-off protein on cellulose. Experiments were performed according to Figure S6. Standard derivation was calculated using data derived from experimental triplicates.

In order to examine the cellulose-binding properties of CBM-fused molecules, varying amounts of ^125^I-labeled CBM-anti-hCG scFv, solitary anti-hCG scFv, solitary CBM protein and solitary antibody (IgG) were applied to cellulose, as well as to nitrocellulose. After several washing steps, radiogram analysis revealed higher amounts of bound CBM, as well as the CBM-scFv, compared to the solitary scFv and the solitary IgG (Fig. 3A). Interestingly, the fusion protein CBM-scFv showed slightly lower binding to cellulose paper compared with the sole CBM molecule (Fig. 3A). At the highest amount of spotted protein (20 µg) more than twice the amount of CBM-scFv remained bound to the cellulose paper, relative to the solitary scFv (Fig. 3A). Contrarily to the cellulose experiments, all proteins spotted onto nitrocellulose showed only minor differences in their binding-ability towards nitrocellulose, independent of applied protein amounts (Fig. 3C). Interestingly, the CBM-scFv reached comparable retention on cellulose as the solitary scFv on nitrocellulose (Figure S5). With respect to the non-identical molecular size of the different proteins, the number of cellulose-bound CBM (19.7 kDa), CBM-anti-hCG scFv (48.7 kDa) and solitary scFv (27.5 kDa) molecules was analyzed. We found that the fusion of CBM to the scFv (CBM-scFv) improved the molar binding capacity (pmol/cm^2^), in relation to the binding capacity of the non-fused scFv. Approximately twice the number of CBM-anti-hCG scFv molecules remain bound onto the cellulose, compared to the solitary scFv (Fig. 3B). Furthermore, the total proportions of bound and washed-off IgG, solitary CBM, solitary scFv and CBM-scFv was analyzed. While the majority of solitary IgG and the solitary scFv molecules was washed away (76–81.2%), significantly larger proportion of CBM-scFv and the solitary CBM remain bound to the cellulose (51.5–60.4%) (Fig. 3D). Comparing the bound protein proportions of the solitary scFv with the CBM-scFv we found that a 2.7-fold amount of CBM-scFv remained bound to the cellulose (Fig. 3D).

### Generation and comparison of CBM-assisted and non-CBM-assisted lateral flow assays

**Figure 4:**
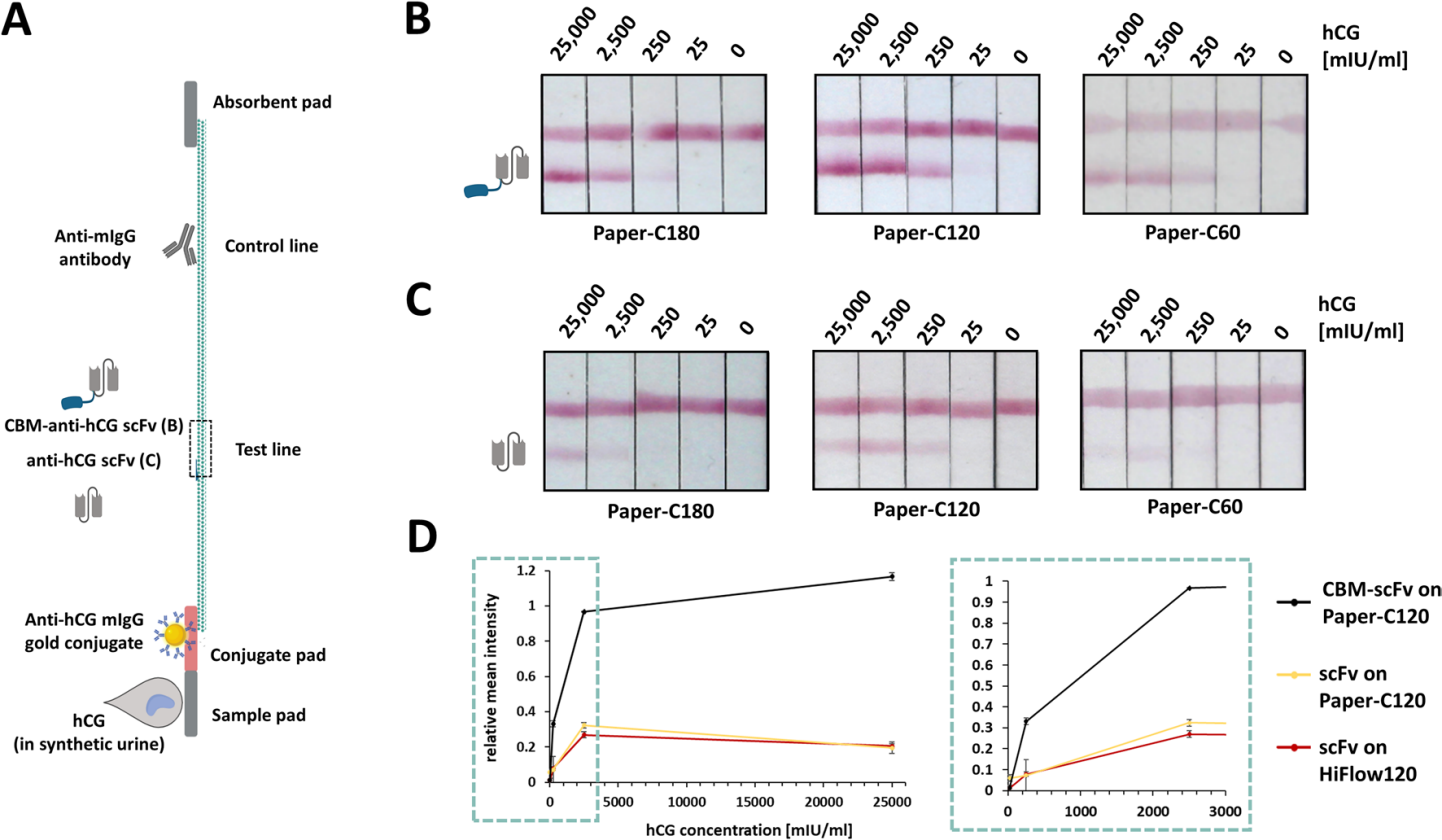
Cellulose-based LFAs for the detection of hCG using different cellulose-based papers (C180, C120, C75). (**A**) Experimental setup for the LFA for pregnancy detection using (**B**) CBM-anti-hCG scFv or (**C**) the corresponding solitary anti-hCG scFv. 150 µl of sample was applied to the sample pad, containing 150 µl synthetic urine with varying concentrations of hCG. Sample compositions are listed in Table S1. (**D**) Analysis of the mean LFA test line intensity normalized to the respective control line intensity. LFA experiments were performed in duplicates. LFAs used for analysis are shown in Figure S9B. Error bars represent the standard derivation.

For verification of the generic applicability of CBM-fused detection antibodies, pregnancy LFA devices were generated using the CBM-anti-hCG scFv, as well as the solitary anti-hCG scFv (Fig. 4A). Those two proteins were printed separately on three different lab-engineered cellulose-papers, differing in their flow characteristics. According to Schabel and coworkers the fluid flow characteristics of lab-engineered paper were modulated by varying the effective specific energies while refining the cotton linters pulp as well as fractionating the fibers before paper sheet production^30^. This resulted in fabrication of papers with capillary flow rates of approximately 60 s/4 cm, 120 s/4 cm and 180 s/4 cm (named C60, C120, C180), representing suitable paper samples for the comparison with classical NC membranes (HiFlow75, HiFlow120, HiFlow180). Lateral flow test devices were assembled and subsequently synthetic urine samples, spiked with varying amounts of hCG were applied. All CBM-assisted lateral flow assays (Fig. 4B) showed an enhanced sensitivity compared to the respective lateral flow test device functionalized with the solitary scFv (Fig. 4C). Furthermore, the CBM-assisted lateral flow assays demonstrated sensitive detection at low hCG concentrations (25 to 250 mIU/ml), independent of the used cellulose-paper. The LFAs, functionalized with the solitary scFv showed less sensitive hCG detection, even though the combination of the solitary scFv and the C120 paper enabled the detection of 250 mIU/ml hCG (Fig. 4C, Paper-C120). However, the intensity of the test line varied depending on the varying flow rate of the used cellulose paper (Fig. 4B,C). Furthermore, we analyzed the relative test line intensities of CBM-anti-hCG scFv and anti-hCG scFv functionalized LFAs (Fig. 4D). The CBM-scFv functionalized LFAs demonstrated a 4.6-fold higher test line intensity compared to the LFAs with the solitary scFv, when applying 250 mIU/ml hCG. Since the lateral flow assays with the C120 paper showed the most sensitive detection (25 mIU/ml hCG) and the best visible test line intensity (Fig. 4B, Paper-C120). For this reason, all following lateral-flow tests were generated using the C120 paper. Additionally, we performed classical LFAs using different NC membranes (HiFlow180, HiFlow120, HiFlow75) comparing the performance of the solitary anti-hCG scFv on NC with the CBM-anti-hCG scFv on cellulose papers (Figure S7). We found that the anti-hCG scFv functionalized NC-LFAs demonstrated sensitive detection of 250 mIU/ml hCG, which is similar to the CBM-anti-hCG scFv functionalized cellulose LFA. However, the LFAs using HiFlow 75 NC membrane (Figure S7B) showed less sensitive hCG detection, while the LFAs using C60 paper (Figure S7C) were able to detect 250 mIU/ml hCG.

**Figure 5:**
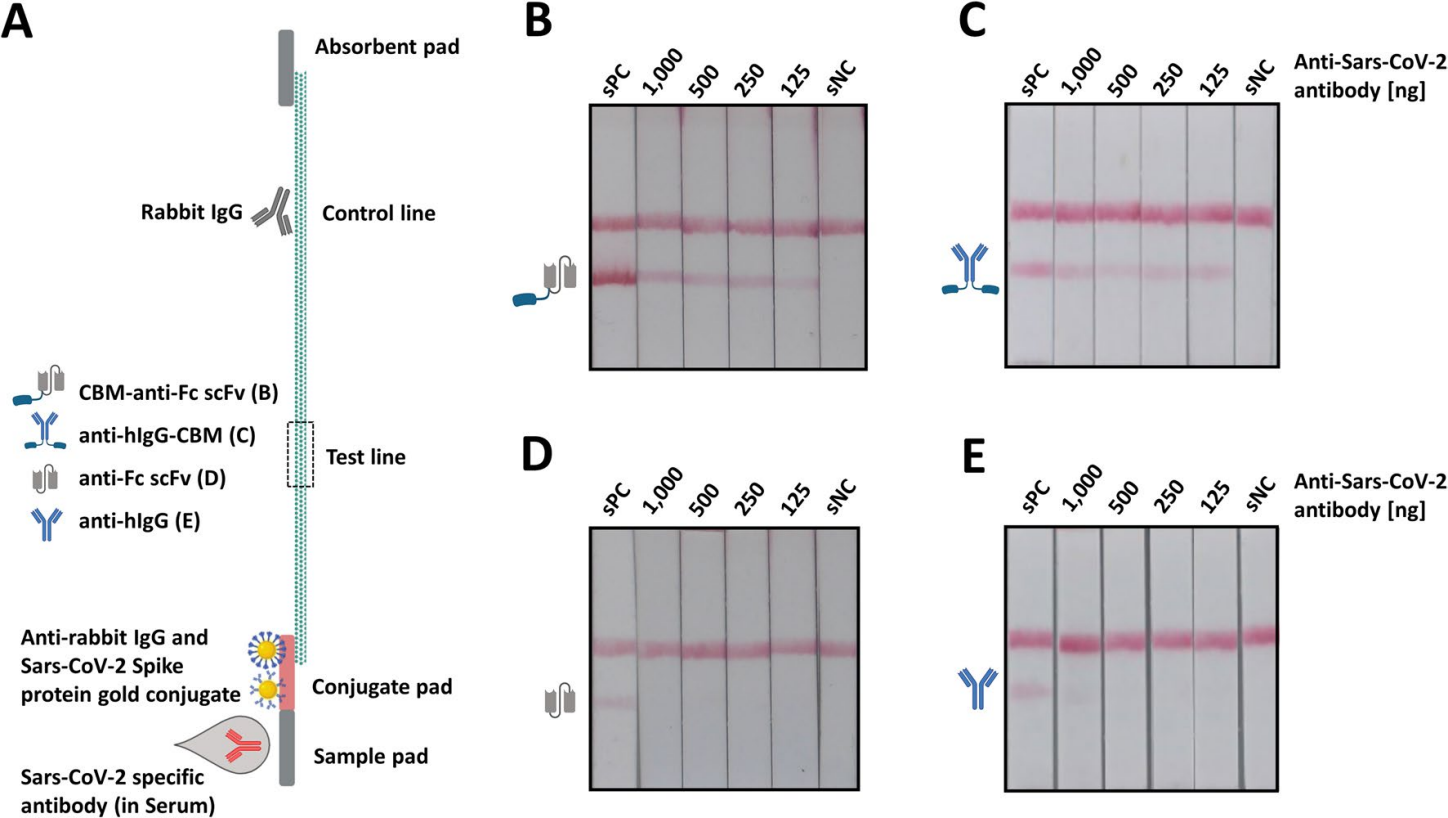
Cellulose-based lateral flow assays for the detection of SARS-CoV-2 specific antibodies in serum samples. (**A**) Experimental setup for the Covid19 antibody LFA using (**B**) CBM-anti-Fc scFv, (**C**) anti-hIgG-CBM, (**D**) anti-Fc scFv or (**E**) anti-hIgG functioning as detection antibody, printed on the test line area. 150 µl of sample was applied to the sample pad, containing 130 µl of TBS buffer,20 µl of human serum and different amounts of anti-SARS-CoV-2 spike antibody. Sample compositions are listed in Table S2.

Inspired by these findings, we generated LFAs for the detection of SARS-CoV-2 specific antibodies by the implementation of CBM-fused antibodies in two different LFA setups (Fig. 5A). The first batch of lateral flow tests was either functionalized with the CBM-anti-Fc scFv or with the solitary anti-Fc scFv, respectively. A second batch of lateral flow tests was functionalized with the full-length IgG multiclonal antibody constructs anti-human IgG or the equivalent IgG-CBM fusion variant. Samples containing different amounts of anti-SARS-CoV-2 S1 specific antibody, mixed with a constant amount of human serum (20 µl) were applied to the lateral flow test strips (Table S2). The CBM-scFv-functionalized Covid19 antibody test demonstrated sensitive detection of 125 ng SARS-CoV-2-specific antibody (Fig. 5B), while the solitary scFv-functionalized LFA only showed a fade test line when applying 1 µg of Sars-CoV-2-specific antibodies, lacking additional serum (Fig. 5D, sample ID: sPC). The lateral flow test strips functionalized with anti-hIgG-CBM (Fig. 5C) showed comparable sensitivity to the CBM-scFv LFA, detecting even low amounts of SARS-CoV-2 specific antibodies. The sole anti-hIgG was able to detect 500 ng of specific antibodies (Fig. 5D), resulting in a fade test line, almost impossible to recognize by naked eye. Together, all CBM-assisted LFAs demonstrated improved sensitivity, compared to LFAs without CBM-fused antibodies. The control line of all performed lateral flow assays showed a strong comparable signal, indicating an accurate assay procedure (Figs. 4, 5, Figure S7, Figure S9). Furthermore, the enhanced test line signal of CBM-assisted LFAs resulted in a simplified visual readout.

## Discussion

With the rapid growth of the world population and emerging global healthcare challenges, the need of point-of-care diagnostic tools, especially lateral flow assays, increases permanently. At the same time issues about the biodegradability and the sustainable use of resources regarding the development and manufacturing of LFAs must be addressed. Here we present a tool to further expand the performance of detection reagents used in the context of cellulose-composed materials. Our platform is based on the cellulose-binding characteristics of the CBM3a molecule, expressed as an antibody-fusion or antibody fragment-fusion protein (IgG-CBM and CBM-scFv). The CBM-fusion proteins showed enhanced performance in the context of a cellulose-based LFAs (Fig. 4D), demonstrating sensitive detection of either the pregnancy hormone hCG in synthetic urine or SARS-CoV-2 specific antibodies present in human serum. Since the CBM-fused antibodies demonstrated improved performance in two different lateral flow test setups (testing urine and serum containing samples) our newly developed tool has the potential to ultimately shift the focus from standard nitrocellulose membranes towards implementation of more sustainable cellulose-composed papers.

The presented cellulose binding studies, proved the enhanced spatially retention of CBM and CBM-fused antibody fragments, resulting in a cellulose binding capacity of CBM-fused scFv comparable to the nitrocellulose binding capacity of the solitary scFv (Figure S5). Furthermore, we found comparable sensitivities, regarding hCG detection using NC-based LFAs (functionalized by anti-hCG scFv) or cellulose-based LFAs (functionalized by CBM-anti-hCG scFv) (Figure S7). This indicates that cellulose-based LFAs could reach similar sensitivities compared to common nitrocellulose-based LFAs, when using CBM-fused detection reagents, which is in accordance with recently published data^24^. Furthermore, we observed a dramatic loss of sensitivity of the solitary detection antibodies and antibody fragments on cellulose-based LFAs, compared to the CBM-fused counterparts (Fig. 5). The comparison of CBM-assisted and non-CBM-assisted Covid-19 antibody LFA (Fig. 5) revealed a substantially stronger difference in sensitivity in relation to the LFA for pregnancy detection (Fig. 4). This effect originates most likely from the presence of competing serum antibodies non-specific for SARS-CoV-2 S1 present in human serum samples, while the detection of hCG in the urine sample is not subjected by this kind of binding competition.

The enhanced LFA detection sensitivity is most likely driven by the spatial retention of the CBM-fused detection agents (Figs. 2, 3). However, an additional positive effect could derive from the binding of the CBM domain to the cellulose which likely results in a beneficial, more accessible orientation of the fused detection antibody. This assumption can be supported by oriented site-directed covalent protein-cellulose conjugation, while random, non-site-specific protein-conjugation could result in unfavorable orientation and accessibility and therefore leading to a partial loss of functionality^18^. In this study we used 40 nm gold nanoparticles conjugated to the SARS-CoV-2 Spike protein. Our results show that the use of gold nanoparticle conjugates can provide sensitive detection of two different analytes (hCG and SARS-CoV-2 specific antibodies) using CBM-fused detection antibodies, applied onto a cellulose paper. However, it has been reported that the use of lanthanide-doped nanoparticles can lead to enhanced sensitivities in the context of lateral flow immunoassays^31^. Hence, application of lanthanide-doped nanoparticles combined with CBM-assisted cellulose-based lateral flow assays could further extend the field of application. The CBM-fusion proteins in this study were generated by genetic fusion to the respective detection antibody or antibody fragment and produced under serum-free conditions, contributing to the sustainability of the application. This option is intrinsically available when using sequence defined antibodies generated with modern in vitro antibody discovery platforms^28^. Nevertheless, long-time validated and well characterized detection antibodies mostly originate from hybridoma clones, that are often lacking protein sequence information. Genetic fusion in this case would require a hybridoma sequencing. To overcome this drawback, direct site-specific conjugation of CBM domains to native antibodies can be considered using a variant of microbial transglutaminase that selectively addresses Gln295 at the antibody Fc as ligand attachment site^32^.

In our study, we used cellulose-only composed paper lacking any chemical modification, exhibiting favorable biodegradability and providing a resource-saving alternative to nitrocellulose-based LFAs^16,33^. Furthermore, flexible cellulose papers can be easily processed using roll-to-roll manufacturing, while common nitrocellulose membranes have a fragile membrane structure making it the most critical material in the LFA^34,35^. This property of NC membranes decelerates the LFA production line and causes a high amount of maculature. With respect to the nearly unlimited cost-friendly resource (cellulose), the relatively simple production and rapid processing of cellulose-papers our approach could ultimately accelerate the mass-production of LFAs and thus contribute to the field of pandemic preparedness^36^.

In summary, we report a strategy to improve the performance of cellulose-based LFAs using detection antibodies directly fused to the carbohydrate-binding module 3a of *Clostridium thermocellum*. Starting with cellulose binding studies, we demonstrated the applicability of CBM-fused antibodies and antibody fragments by implementation into two model LFAs, using CBM-fused scFvs as well as CBM-fused full-length antibodies. In conclusion, we believe that our novel approach could enable the generation of cellulose-based lateral flow test devices, including serologic LFAs and encourage a more frequent use of cellulose instead of nitrocellulose, ultimately resulting in more sustainable and resource-saving POC diagnostics.

## Materials and methods

### Generation of detection antibodies and CBM-fusion proteins

The DNA amplicon, coding for the CBM3a protein followed by a P/T-linker (Table S3), the respective scFv (anti-hCG scFv or anti-Fc scFv) and a StrepTagII was cloned in the bacterial expression vector pET30a via golden gate assembly^27^. The same was performed for the solitary anti-hCG and anti-Fc scFv, likewise fused to a StrepTagII coding sequence. The resulting expression plasmids were used to transform Shuffle T7 Express *E. coli* cells, containing a pLysS Erv1p/DsbC plasmid. The expression of the CBM-fused scFvs and solitary scFvs was performed in SB medium (yeast extract 20 g/L; peptone ex casein 32 g/L; NaCl 5 g/L; NaOH 1 N 5 mL) with a final concentration of 0.1% (w/v) arabinose and 1 mM isopropyl β-D-thiogalactosid (IPTG) at 30 °C, 180 rpm for approximately 16 h, according to^27,37^. Cells were pelleted by centrifugation and subsequently lysed by sonification. Biotinylated host cell proteins were blocked using avidin (Iba Lifescience) according to the manufactures protocol. The sterile-filtered cell lysate was applied on a StrepTactin XT high capacity column (Iba Lifescience) using an ÄKTA Start FPLC system. The purified proteins were transferred to a dialysis hose (MWCO 3 kDa) and two dialysis steps using phosphate-buffered saline (PBS) (137 mM NaCl, 2.7 mM KCl, 10 mM Na_2_HPO_4_, 1.8 mM KH_2_PO_4_) (pH 7.4) were performed. Anti-human IgG-Fc multiclonal antibody VH and VL genes were cloned respectively in the mouse IgG2a variant of the pCSEH1c (heavy chain) and pCSL3k/3 l (light chain kappa/lambda) mammalian expression vectors, via golden gate assembly^38^. For the production of full-length IgG-CBM molecules, the heavy chain vectors were modified by addition to the 3′ CH3 sequence of the DNA encoding for the N-terminal P/T-linker followed by the CBM3a protein and hexameric histidine tag (Table S3). Antibody light chain expression vectors together with CBM3a-fused or -free heavy chain vectors were used to co-transfect mammalian EXPI293F cells (Thermo Fisher Scientific) as previously described^39^. After 6–7 days post transfection, the production supernatant was harvested and secreted antibodies purified via Protein A affinity chromatography.

### Materials and procedure of the substrate binding studies

The protein binding capacity of nitrocellulose membrane and cellulose paper (Cotton linters) was analyzed by ^125^I radiolabeled analytics. Substrate-binding of the solitary scFv, the CBM-scFv and the CBM3a (CZ00411, NZYTech Ltd) as well as a full-length antibody (Cetuximab, Merck) to unbacked NC membrane Hi-Flow Plus HF180 (HF180UBXSS), Whatman Grade 41 (WHA1441042, Sigma-Aldrich) was compared. 30 µg of each protein was radio-iodinated with ^125^I radioisotope using Pierce pre-coated iodination tubes (28,601, Thermo Fisher Scientific) applying the Chizzonite indirect method for iodination to avoid oxidative stress-depending damages of proteins^40^. Iodination was performed in PBS (137 mM NaCl, 2.7 mM KCl, 10 mM Na_2_HPO_4_, 1.8 mM KH_2_PO_4_ pH 7.4) with 7.4 MBq of radioactivity used per sample. CBM3a was purchased in radio-iodination incompatible buffer and thus applied to Zeba Spin Desalting Columns (89,882, Thermo Fisher Scientific) for buffer exchange to PBS + 3.5 mM CaCl_2_ + 25% (v/v) glycerol. After iodination samples were purified from free ^125^I by means of Zebra Spin Desalting Columns using TBS (50 mM Tris–HCl pH 7.5, 150 mM NaCl) + 25% glycerol. Protein buffer was supplemented with additional 3.5 mM CaCl_2_ to preserve the cellulose binding activity of the CBM3a protein domain^41^. Specific radioactivity of the resulting purified post-iodination mixtures was measured using ^125^I automatic gamma counter (2470 WIZARD, Perkin Elmer). The protein concentration was determined via Pierce 660 nm Protein Assay Kit (22662, Thermo Fisher Scientific). For the CBM binding studies (Fig. 2A) 0.5 µg of isotope labeled CBM3a was spotted at the area A (3 cm from base) of a 12 cm Whatman Grade 41 or Hi-Flow Plus HF180 strip. After drying (2 h, 37 °C) 100 µl of TBS buffer, containing different additives (0.1% (w/v) BSA, 0.05% (w/v) Tween-20, 1% (w/v) casein) was applied at the base of each strip. The individual strips were dried and analyzed via phosphor imaging. The experimental setup is depicted in Figure S3. For the quantitative analysis of the binding properties of CBM3a towards cellulose or nitrocellulose (Fig. 2B), 0.5 µg of isotope labeled CBM3a was spotted onto 0.6 cm discs, consisting of Whatman Grade 41 or Hi-Flow Plus HF180. After drying (2 h, 37 °C) each disc was transferred to a 1.5 ml reaction tube, flowed by 3 consecutive washing steps (10 min, 800 rpm, RT) using 300 µl of TBS buffer containing different additives (0.1% (w/v) BSA, 0.05% (w/v) Tween-20). The individual discs were dried and analyzed via phosphor imaging. The experimental setup is depicted in Figure S4. For cellulose and nitrocellulose binding studies and the wash-off experiments of the CBM-scFv fusion protein (Fig. 3), the solitary scFv, a full-length antibody, and the CBM3a molecule, spotting mixtures containing different amounts of the respective protein (0.1, 0.5, 1, 2.5, 5, 10, 20 µg) were spiked with 100 Bq of the respective ^125^I radio-iodinated protein. 6 µl of each spotting mixture were dropped in triplicates onto a wax printed dot-blot array (spot Ø of 2.5 mm) on either unbacked NC membrane or lab-engineered paper (75 g/m^2^) placed on an array grid. For wax array dot blot coating of either cellulose paper or nitrocellulose membrane, a Xerox ColorQube 8580 printer was used. The wax of the printed arrays was melted 3 min at 120 °C in a heating chamber to assure homogeneous heating. After incubation for 2 h at 37 °C the arrays were exposed to phosphor imaging plates as input control. The phosphor imaging plates were scanned with a phosphor imager (Typhoon FLA 7000, GE) and afterwards each array spot was washed three-times with 20 µl washing buffer (TBS + 0.1% (w/v) BSA + 0.05% (w/v) Tween-20). The washing drops were sucked off from the bottom of the NC membrane or paper array by a wicking-blot paper. Arrays were dried 30 min at 40 °C and again were exposed to phosphor imaging plates and subsequently analyzed via phosphor imaging. Image normalization during phosphor imaging was assured by specified 10 Bq drops. The experimental setup is depicted in Figure S6.

### Materials for the generation of cellulose-based lateral flow tests devices

The anti-hCG gold conjugate (62-H25C) as well as human chorionic gonadotropin (hCG, 30–1349) was purchased from Fitzgerald Industries International. Anti-mouse IgG (goat) antibody (MFCD001626638, Sigma-Aldrich) was used, for the control line of the pregnancy lateral flow test strips. For the conjugation of the SARS-CoV-2 antigen to gold nanoparticles the Gold Conjugation Kit (40 nm, 20 OD) (ab154873, abcam) and the SARS-CoV-2 Spike/RBD protein (His Tag) (40150-V08B2, Hölzel) was utilized. Gold nanoparticle conjugation of the SARS-CoV-2 Spike/RBD protein was performed according to the manufacturers protocol using 3 µg SARS-CoV-2 Spike/RBD protein for one reaction mixture, finally resulting in 500 µl (OD 2) of protein-gold conjugate. Anti-SARS-CoV-2 Spike antibody (Ab01580-10.0, IgG1, Absolute Antibody) served as a positive control analyte for the detection of SARS-CoV-2 specific antibodies. IgG from rabbit serum (I8140-10MG, Sigma-Aldrich) as well as anti-rabbit IgG (H + L) 40 nm gold conjugate (BBI solutions) served as reagents for the control line of the Covid-19 antibody LFA. Synthetic urine (S-020-50ML, Surine negative urine control) and human serum (S1-100ML, normal) was purchased from Sigma-Aldrich.

### Manufacturing of cellulose-based lateral flow test devices

For detection antibodies striping onto NC membrane or cellulose paper a System 30 M 3D-printer (Hyrel) equipped with a syringe-based print head was used. The print head was a SDS-5 Extruder (Hyrel) customized to be equipped with a microliter syringe (Hamilton type 1710 N, article 81075, 100 µl volume, 22 s gauge needle, 51 mm needle length, blunt tip). The needle was additionally bent to have a cured shape and softly touching the substrates at an angle of about 30°. The substrates were placed on a vacuum table with a porous and smooth surface made of sintered ceramic particles. Surface was covered with self-adhesive plastic cards leaving a slot to place the substrates. Printing was done at 10 mm/s print speed by dragging the needle tip in soft touch over the substrates surface. Printing was started and ended 3 mm before and after the substrate, to avoid inhomogeneity at starting and ending be applied onto the substrate. See Figure S8 for details of the printing setup. The cotton linters fibers for lab-engineered papers C180 and C120 were refined in a Voith LR 40 laboratory refiner with a 3/3-1.0-60 conic milling set using effective specific energies of 75 kWh/t for C180 and 225 kWh/t for C120. For production of C120 paper the cotton linters pulp was fractionated with a Haindl-McNett fractionator using the R200 filter with a mesh size of 0.075 mm. Lab-engineered paper C60 was made out of untreated cotton linters pulp. Paper sheets of 75–78 g/m^2^ grammage were produce on a conventional Rapid-Koethen handsheet mould (DIN EN ISO 5269–2). The capillary flow rates (s/4 cm) were measured by applying water to the bottom of a 4 cm test strip and measuring the time until reaching the 4 cm mark. Test line solutions containing 1 mg/ml of solitary scFv, CBM-scFv, anti-hIgG or anti-hIgG-CBM in 10 mM MES (pH 6.5) containing 3 mM CaCl_2_ and control line solution containing 1 mg/ml goat anti-mouse capture antiserum (M8890, Merck) or rabbit IgG were striped at 0.1 µl/mm flow to yield 0.5 µg protein per LFA strip. Membrane and paper substrates were dried 2 h at 37 °C. Anti-hCG gold conjugates (OD 2) in 5% (w/v) trehalose were applied onto glass fiber conjugate pads (GFCP203000 GFDX203000, Merck Millipore) and dried overnight at room temperature. Anti-rabbit IgG (goat) gold conjugate (OD2) and Sars-CoV-2 Spike protein gold conjugate (OD 2) both in 5% (w/v) trehalose was mixed and applied onto glass fiber conjugate pads and dried overnight at room temperature. Cellulose pads (CFSP 223,000, 20 × 30 cm sheets, Merck Millipore) were used to manufacture the sample and absorbent pads. Sample pads were soaked in Tris–HCl (pH 7.4), 1% (w/v) Tween-20, 0.75% (w/v) BSA and dried overnight, followed by 2 h at 37 °C. Sample pads, absorbent pads, conjugate pads and paper substrates were sealed and evacuated in aluminum foil bags and stored at 4 °C. Adhesive backing cards (HF000MC100, Merck Millipore) were utilized a supporting unit for the assembly of lateral flow test devices. Cellulose-paper substrates, functionalized with the respective antibody or CBM-fused antibody as well as the corresponding conjugate pads were assembled with cellulose sample pads (CFSP223000, Merck Millipore; treated with 10 mM Tris–HCl pH 8.2, 1% (w/v) Tween 20, 0.75% (w/v) BSA) and absorbent pads (CFSP223000, Merck Millipore) into a classical lateral flow assay setup (Figs. 4A, 5A).

### Lateral flow assay procedure and analysis

The scFv and CBM-scFv functionality in lateral flow test devices was analyzed by using a hCG concentration series of 0, 25, 250, 2500 and 25,000 mIU/ml hCG spiked in synthetic urine (S-020, Cerilliant) (Table S1). 150 µl of each concentration were applied onto the center of the LFA sample pad, passing the conjugate pad by capillary action, migrating over the paper substrate and finally being wicked by the absorbent pad. The signal intensity of each LFA strip and control line was analyzed visually after 20 min using a reflex camera (EOS 600D, Canon). Covid-19 antibody LFA device functionality was analyzed using 20 µl of human serum with varying concentrations and total amount of anti-SARS-CoV-2 Spike protein antibody (Ab01580-10.0, IgG1, Absolute Antibody) (Table S2). 130 µl of sample buffer (Tris pH 7.4, 150 mM NaCl) was added and the resulting analyte solution was pipetted onto the sample pad. The test result was analyzed visually after 20 min using a reflex camera (EOS 600D, Canon). Quantitative analysis of LFAs was performed using ImageJ (version 1.35e). The mean test line intensity was normalized to the mean intensity of the respective control line. All values were normalized to the mean test line intensity of the negative control LFA (without analyte). Analysis and calculation of standard derivation was performed using duplicate LFA results.

## Supplementary Information

Supplementary Information.
