# Supplementary material for: Carbohydrate binding module-fused antibodies improve the performance of cellulose-based lateral flow immunoassays
Source: Sci Rep. 2021 Apr 12;11:7880. doi: 10.1038/s41598-021-87072-7 (PMC8042022; doi:10.1038/s41598-021-87072-7)
Supplement: Supplementary file 1 — Supplementary Information. [file 41598_2021_87072_MOESM1_ESM.pdf]

Supplemental

Carbohydrate binding module-fused antibodies improve the performance of cellulose-based lateral flow immunoassays

Adrian Elter[1,2], Tina Bock[2,3], Dieter Spiehl[2,4], Giulio Russo[5,6], Steffen C. Hinz[1,2], Sebastian Bitsch[1,2], Eva Baum[1,2], Markus Langhans[7], Tobias Meckel[2,7], Edgar Dörsam[4], Harald Kolmar*[1,2] and Gerhard Schwall*[2,3]

[1]Institute for Organic Chemistry and Biochemistry, Technical University of Darmstadt, Alarich-Weiss-Strasse 4, D-64287 Darmstadt, Germany

[2]Merck Lab @ Technical University of Darmstadt, Alarich-Weiss-Strasse 8, D-64287 Darmstadt, Germany

[3]Sustainability, Science & Technology Relations, Merck KGaA, Frankfurter Strasse 250, D-64293 Darmstadt, Germany

[4]Institue of Printing Science and Technology, Technical University of Darmstadt, Magdalenenstrasse 2, D-64289 Darmstadt, Germany

[5]Department of Biotechnology, Technical University of Braunschweig, Spielmannstrasse 7, D-38124 Braunschweig, Germany

[6]Abcalis GmbH, Inhoffenstrasse 7, D-38124 Braunschweig, Germany

[7]Macromolecular Chemistry and Paper Chemistry, Technical University of Darmstadt, Alarich-Weiss-Strasse 8, D-64287 Darmstadt, Germany

*Corresponding authors Harald Kolmar (Email: *Harald.Kolmar@TU-Darmstadt.de*) and Gerhard Schwall (Email: *Gerhard.Schwall@Merckgroup.com*)

Keywords

Lateral flow assay, point-of-care diagnostics, Covid-19 antibody test, pregnancy detection, carbohydrate-binding module, cellulose, sustainability, SARS-CoV-2

Supplementary Figures

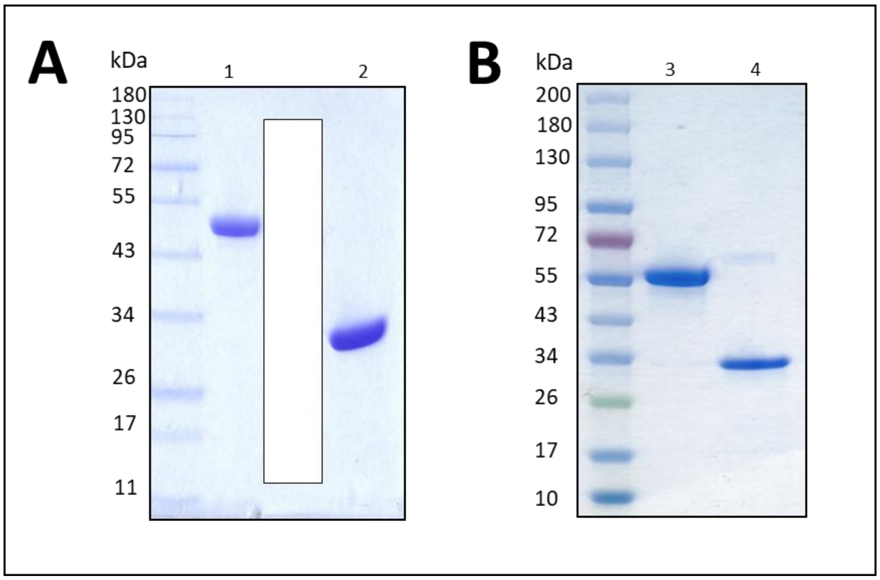

Figure S1. SDS-PAGE and Coomassie staining analysis with the purified protein samples used for the generation of LFTs for the detection of (A) hCG and (B) SARS-CoV-2 specific antibodies. (1) CBM-anti-hCG-scFv; (2) anti-hCG scFv; (3) CBM-anti-Fc scFv; (4) anti-Fc scFv. (**A**) The protein ladder (Blue Prestained Protein Standard Broad Range; New England Biolabs) and (**B**) Color Prestained Protein Standard Broad Range; New England Biolabs) was used.

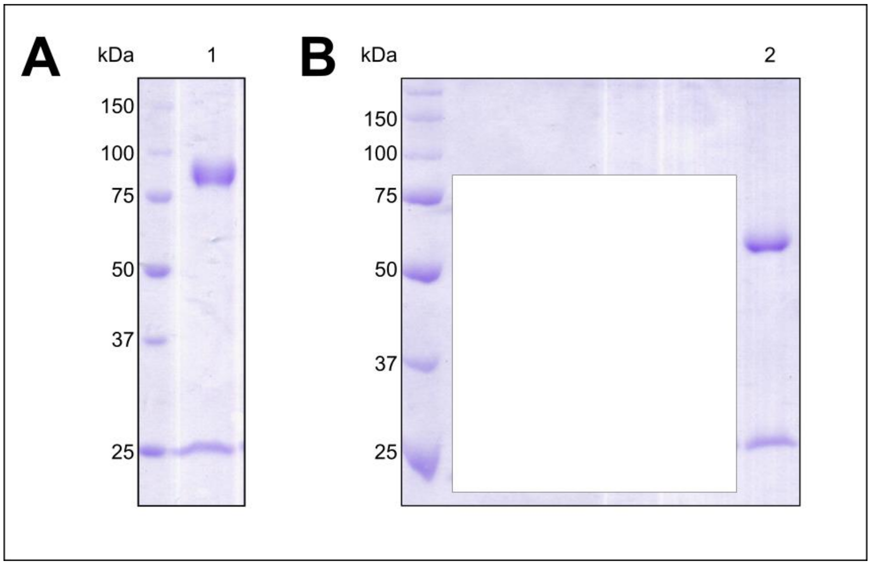

Figure S2. SDS-PAGE and Coomassie staining analysis of protein A affinity purified full-length IgG and IgG-CBM fusion protein samples used for the generation of LFTs for the detection of human SARS-CoV-2 specific antibodies. (**A**) representative mIgG2a-CBM-HIS anti-human IgG; (**B**) representative mIgG2a anti-human IgG. The protein ladder Precision Plus Protein Unstained Standards (Bio-Rad) was used.

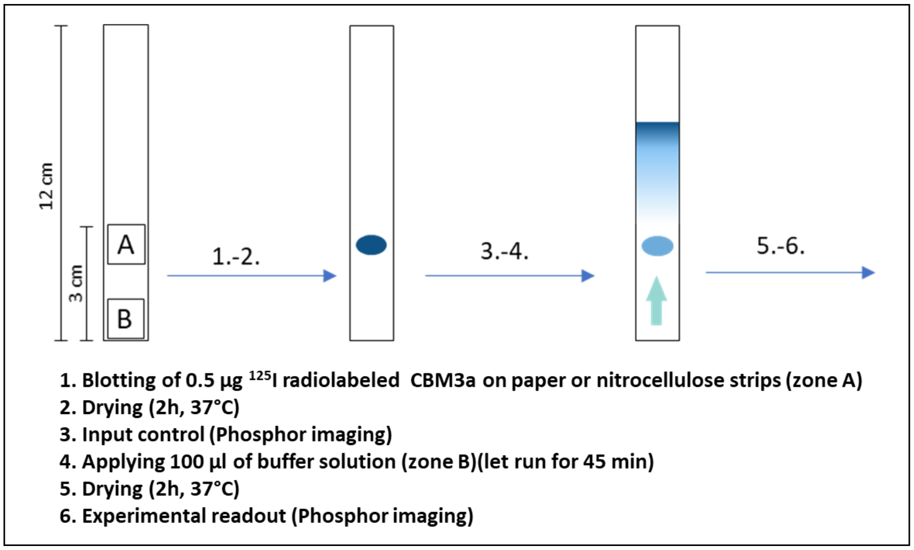

Figure S3. Experimental setup and procedure, for the spatially resolved analysis of CBM binding on cellulose and nitrocellulose. Detailed description can be found in the materials and methods section.

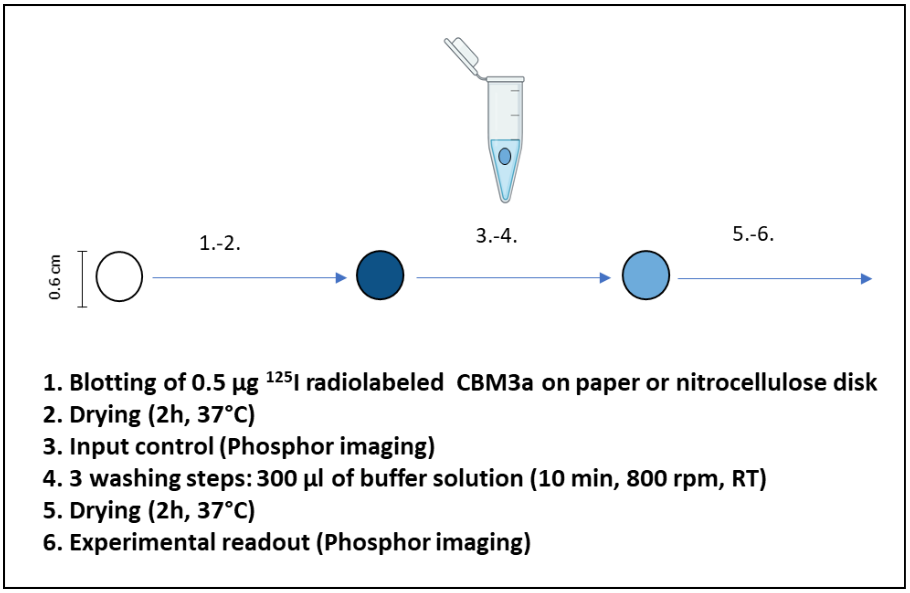

Figure S4. Experimental setup and procedure, for the quantitative analysis of CBM binding on cellulose and nitrocellulose. Detailed description can be found in the materials and methods section.

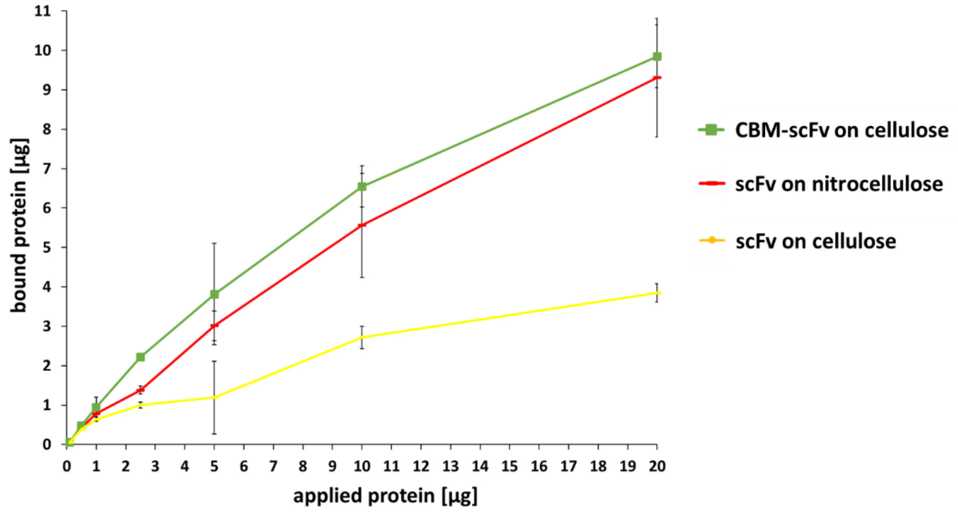

Figure S5. Quantitative analysis of the protein binding capacity of CBM-scFv and the corresponding solitary scFv on cellulose in comparison to the binding capacity of the solitary scFv on nitrocellulose. Experiments were performed according to Figure S6. Standard derivation was calculated using data derived from experimental triplicates.

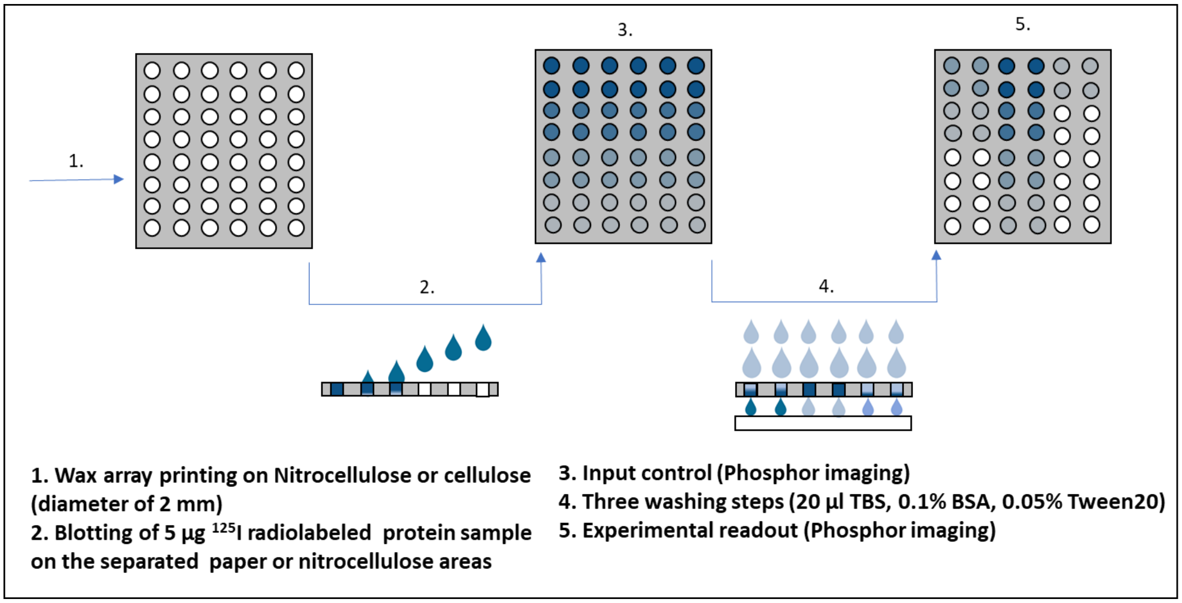

Figure S6. Experimental setup and procedure, for the quantitative analysis of CBM, CBM fused molecules and non-fused molecules binding on cellulose and nitrocellulose. Detailed description can be found in the materials and methods section.

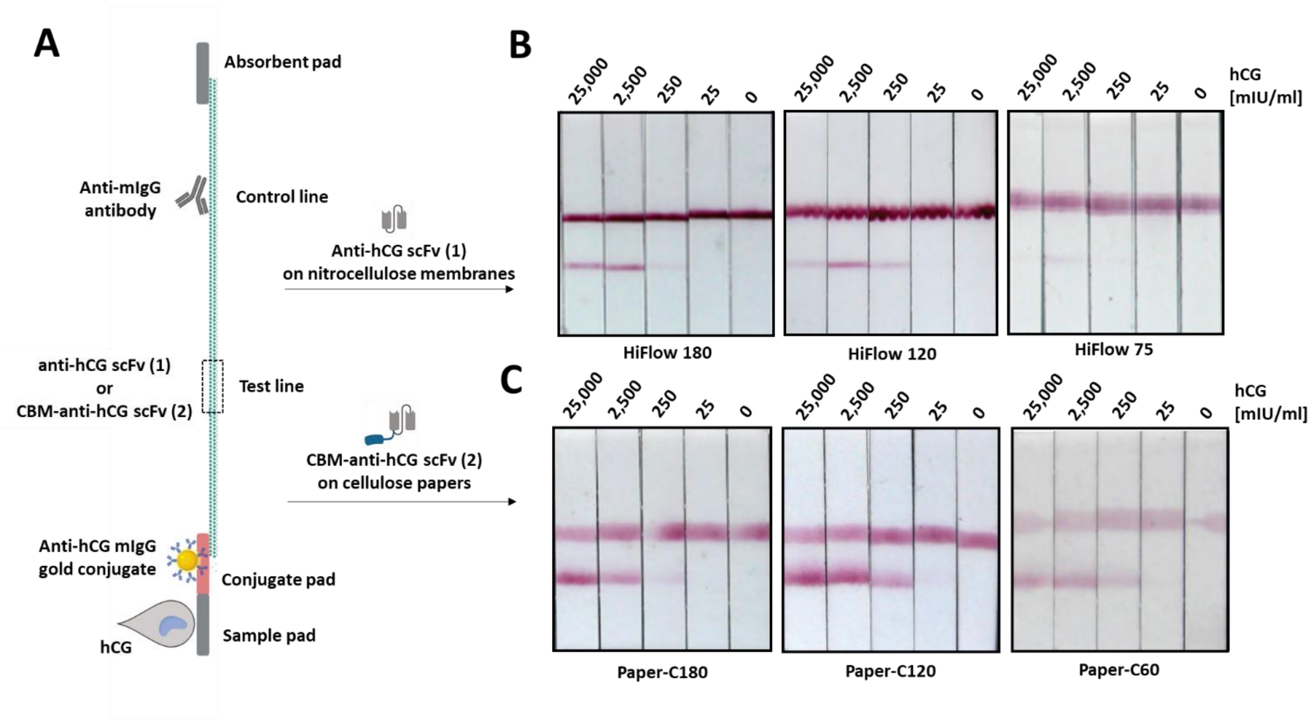

Figure S7. LFTs for the detection of hCG using different cellulose-based papers (C180, C120, C60) and NC membranes (HiFlow180, HiFlow120, HiFlow75). (**A**) Experimental setup for the LFT for pregnancy detection using (**B**) the solitary anti-hCG scFv on NC membranes or (**C**) CBM-anti-hCG scFv on cellulose paper. 150 µl of sample was applied to the sample pad, containing 150 µl synthetic urine with varying concentrations of hCG. Sample compositions are listed in Table S1. LFA experiments were performed in duplicates, all shown in Figure S9.

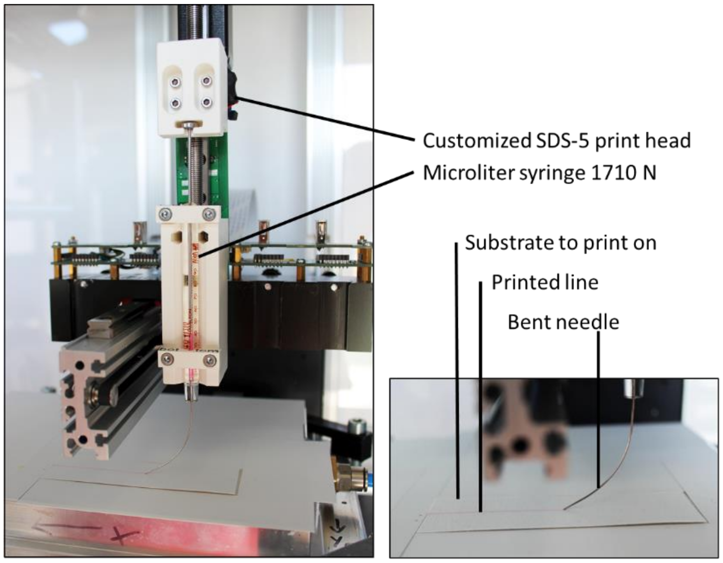

Figure S8. Photography of the printing area of the 3D-printer System 30M used to strip the detection antibodies onto the membrane or paper. The customizes SDS-5 print head loaded with the microliter syringe 1710 N including the bent needle can be seen. As a showcase red colored water (to see the printed line) is printed onto a paper cardboard (to distinguish the thicker substrate from the self-adhesive cards covering the vacuum table). The inset on the lower right shows a detailed photography of the print.

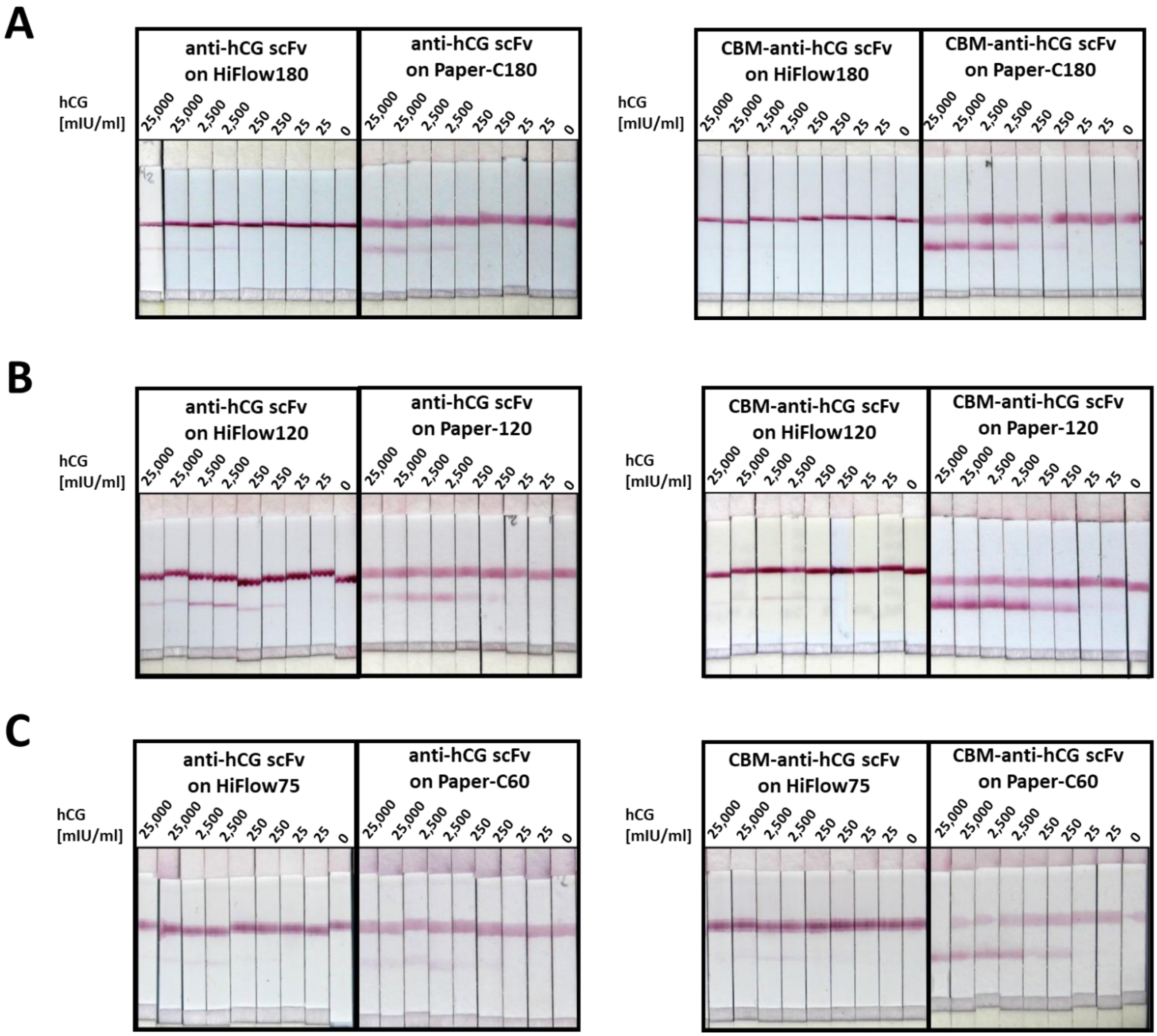

Figure S9. LFTs for the detection of hCG using the CBM-anti-hCGH scFv or the anti-hCG scFv. Different cellulose-based papers (C180, C120, C60) and NC membranes (HiFlow180, HiFlow120, HiFlow75) were used. (**A**) LFTs based on HiFlow180 nitrocellulose membranes or Paper-C180 (**B**) LFTs based on HiFlow120 nitrocellulose membranes or Paper-C120 (**C**) LFTs based on HiFlow75 nitrocellulose membranes or Paper-C60. 150 µl of sample was applied to the sample pad, containing 150 µl synthetic urine with varying concentrations of hCG. Sample compositions are listed in Table S1.

Supplementary Tables

Table S1. Sample composition for the validation of the pregnancy lateral flow assays.

Sample ID	Synthetic urine (hCG negative) [µL]	Human chorionic gonadotropin (hCG) [mIU/ml]	Human chorionic gonadotropin (hCG) [mIU]
**h1**	150	25,000	3,750
**h2**	150	2,500	375
**h3**	150	250	37.5
**h4**	150	25	3.75
**hCG negative control (hNC)**	150	0	0

Table S2. Sample composition for the validation of the Covid-19 antibody lateral flow assays.

Sample ID	anti-Sars-CoV-2 IgG [µg]	Human Serum (7.5 to 22 µg IgG/µl) [µl]	Total IgG [µg]	anti-Sars-CoV-2 IgG [%]
**s1**	1	20	(15 to 44)+1	2.22 to 6.25
**s2**	0.5	20	(15 to 44)+0.5	1.11 to 3.32
**s3**	0.25	20	(15 to 44)+0.25	0.56 to 1.63
**s4**	0.125	20	(15 to 44)+0.125	0.28 to 0.82
**s5**	0.0625	20	(15 to 44)+0.0625	0.14 to 0.41
**s6**	0.03125	20	(15 to 44)+0.03125	0.07 to 0.2
**negative control sample (sNC)**	0	20	15 to 44	0
**Positive control sample (sPC)**	1	0	1	100

Table S3. Protein Sequence information of CBM-scFv and full-length antibodies fused to CBM (IgG-CBM), including linker sequences and purification tag.

Protein	Sequence Information
**CBM-scFv**	ANTPVSGNLKVEFYNSNPSDTTNSINPQFKVTNTGSSAIDL<br>SKLTLRYYYTVDGQKDQTFWSDHAAIIGSNGSYNGITSNVK<br>GTFVKMSSSTNNADTYLEISFTGGTLEPGAHVQIQGRFAK<br>NDWSNYTQSNDYSFKSASQFVEWDQVTAYLNGVLVWGK<br>EPGELGSVVPSTQPVTTPPATTKPPATTIPPSDDPNLEVLFQ<br>GPAS-scFv protein sequence-GSWSHPQFEK
**Full-length antibody-CBM**	Antibody heavy chain protein sequence-<br>GSGNATPTKGATPTNTATPTKSATATPTRPSVPTNTPTNTPA<br>NTPVSGNLKVEFYNSNPSDTTNSINPQFKVTNTGSSAIDLS<br>KLTLRYYYTVDGQKDQTFWSDHAAIIGSNGSYNGITSNVK<br>GTFVKMSSSTNNADTYLEISFTGGTLEPGAHVQIQGRFAK<br>NDWSNYTQSNDYSFKSASQFVEWDQVTAYLNGVLVWGK<br>EPGGSHHHHHH
